# Association Between a Directly Translated Cognitive Measure of Negative Bias and Self-reported Psychiatric Symptoms

**DOI:** 10.1016/j.bpsc.2020.02.010

**Published:** 2022-02

**Authors:** Lucie Daniel-Watanabe, Martha McLaughlin, Siobhan Gormley, Oliver J. Robinson

**Affiliations:** a Institute of Cognitive Neuroscience, University College London, London, United Kingdom; b MRC Cognition and Brain Sciences Unit, University of Cambridge, Cambridge, United Kingdom; c Department of Psychiatry, University of Cambridge, Cambridge, United Kingdom

**Keywords:** Anxiety, Computational psychiatry, Depression, Individual differences, Negative affective bias, Online testing, Structural equation modeling

## Abstract

**Background:**

Negative interpretation biases are thought to be core symptoms of mood and anxiety disorders. However, prior work using cognitive tasks to measure such biases is largely restricted to case-control group studies, which cannot be used for inference about individuals without considerable additional validation. Moreover, very few measures are fully translational (i.e., can be used across animals and humans in treatment-development pipelines). This investigation aimed to produce the first measure of negative cognitive biases that is both translational and sensitive to individual differences, and then to determine which specific self-reported psychiatric symptoms are related to bias.

**Methods:**

A total of 1060 (*n* = 990 complete) participants performed a cognitive task of negative bias along with psychiatric symptom questionnaires. We tested the hypothesis that individual levels of mood and anxiety disorder symptomatology would covary positively with negative bias on the cognitive task using a combination of computational modeling of behavior, confirmatory factor analysis, exploratory factor analysis, and structural equation modeling.

**Results:**

Participants with higher depression symptoms (β = −0.16, *p = .*017) who were older (β = −0.11, *p = .*001) and had lower IQ (β = 0.14, *p < .*001) showed greater negative bias. Confirmatory factor analysis and structural equation modeling suggested that no other psychiatric symptom (or transdiagnostic latent factor) covaried with task performance over and above the effect of depression, while exploratory factor analysis suggested combining depression/anxiety symptoms in a single latent factor. Generating groups using symptom cutoffs or latent mixture modeling recapitulated our prior case-control findings.

**Conclusions:**

This measure, which uniquely spans both the clinical group-to-individual and preclinical animal-to-human generalizability gaps, can be used to measure individual differences in depression vulnerability for translational treatment-development pipelines.

Negative biases have long been implicated in mood and anxiety disorder pathogenesis (1, 2, 3, 4, 5, 6). Modifying negative biases is the theoretical basis for cognitive behavioral therapy as well as a proposed mechanism of action for antidepressant treatment (7). However, for a majority of individuals the first treatment that they try does not work (8,9).

This treatment targeting failure highlights at least 2 problems. First, we lack good measures of individual differences that we can use to tailor personalized treatments. Indeed, the vast majority of cognitive measures of affective bias are validated for group differences rather than individual differences. That is to say, although groups with increased symptomatology show increased negative bias, this does not necessarily mean that an individual within a group’s specific bias levels can predict their individual symptoms or treatment response. Recent work demonstrating that individual levels of emotional bias and subjective symptoms can predict response to antidepressant medication highlights the importance of this (10).

Second, from a personalized treatment development perspective, we lack tasks that can be used in translational treatment-development pipelines. Many studies examining negative bias use tasks that do not have clear analogues in animal models [for example, they require language (4,6)], and as such, early trials of new candidate interventions are not tested against the same measures that are used in humans. This means that we do not know which specific symptoms will benefit from a given intervention and therefore which particular patients the intervention should be given to.

We recently addressed the second limitation by directly translating a measure of negative interpretation bias from rodents (11) (Figure S1A) into humans (12) (Figure S1B). That study demonstrated a significantly greater negative interpretation bias in a group of clinically screened participants who met criteria for mood or anxiety disorders relative to a group of control participants (12). However, the experiment was conducted in a small sample of mixed and comorbid depression and anxiety disorders, and differences were seen in a group-level comparison. It is therefore unclear if this measure is viable as a measure of individual differences in negative bias vulnerability. The present study therefore sought to adapt the task used in our previous study (12) across domains to detect individual differences and then extend it into a large sample of individuals from whom we also collected self-report symptoms. As such, we attempted to validate the model of negative bias translated from rodent models and to demonstrate the generalizability of the stimuli across domains.

We therefore had three primary objectives. First, we wanted to explore and remove sources of between-subject bias within the task to maximize our chances of measuring individual differences in task performance. Second, we wanted to explore factors contributing to individual differences in task performance in a large cross-sectional sample, particularly which specific psychiatric-relevant symptoms/traits contribute to task performance. Finally, having identified relevant traits, we wanted to recapitulate the effect of clinical screening in a large unscreened sample.

## Methods and Materials

### Recruitment

All studies were carried out online using the recruitment platform Mechanical Turk (www.mturk.com) and the Gorilla platform (13) (www.gorilla.sc) for social sciences. Participants were not restricted to any geographic area (which may lead to some unique data features—see the Supplement). To facilitate online data collection, we switched the task from the auditory domain to the visual domain (14). In the first pilot (see the Supplement) we tested 264 participants. Following discovery of between-subject bias we next tested 158 individuals on a different version of the task. This second task version was preferred because, although there was still evidence of a bias, the stimuli in the “orientation” version only varied on a single dimension (line orientation), whereas in the “circle size” version they varied on both area and radius.

A total of 1060 participants (622 male, 434 female, 4 other) were then recruited and tested for the main study. Seventy were excluded because of failure to complete the task correctly (e.g., responding using only 1 button press in the task, or letting the task run without responding), leading to a final sample of *n* = 990 (Table 1) (although findings hold if the missing data is estimated—see the Supplement). Data were collected in batches from April to September 2018.

**Table 1:** Comparison Between Participants Included in the Final Analysis and Those That Were Excluded Because of Failure to Complete the Task

Variable	Final Sample (*n* = 990)				Excluded From Analysis (*n* = 70)			
	Mean	SD	Lower Limit	Upper Limit	Mean	SD	Lower Limit	Upper Limit
Age, Years	34	10	18	76	34	10	22	63
Raven’s	4	3	0	12	3	3	0	9
OCI-R	24	18	0	72	30	19	0	64
SZ	16	9	0	42	20	9	1	51
BDI	15	12	0	56	16	12	0	57
STAI	45	12	20	78	47	11	20	74

Note that including these participants and imputing values for the missing data resulted in identical inference.

BDI, Beck Depression Inventory II; OCI-R, Obsessive-Compulsive Inventory-Revised; Raven’s, Raven’s Progressive Matrices (12-item form); STAI, State-Trait Anxiety Inventory; SZ, Schizotypy short scale.

### Procedure

This study was approved by the University College London Research Ethics Committee (6199/001). Participants were provided with an information sheet and then completed an online consent form. They then completed symptom questionnaires and the task (see below).

### Questionnaires

Participants in the main study completed the Schizotypy short scale (15), the Obsessive-Compulsive Inventory-Revised (OCI-R) (16), the State-Trait Anxiety Inventory (STAI) (17), the Beck Depression Inventory II (BDI) (18), and a 12-item form of Raven’s Progressive Matrices (19) as a measure of IQ.

### Task

**Figure 1:**
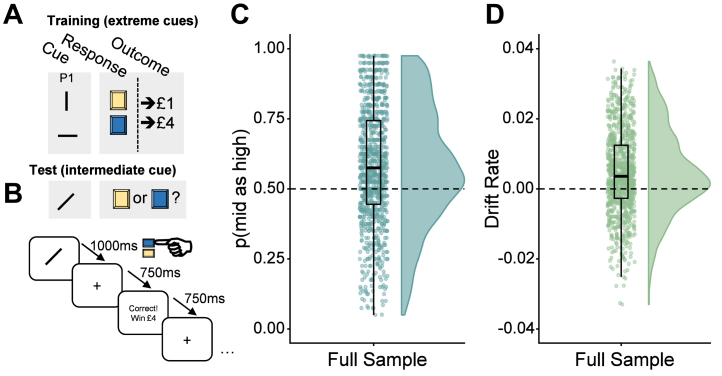
Task schematic and development. **(A)** Participants were first trained on “extreme” cues (vertical or horizontal lines) that had 100% contingencies with high or low reward (hypothetical £1 or £4) contingent on participants pressing the correct button (left or right; represented as blue or yellow in this figure). Following training, participants were presented with intermediate stimuli (angled lines) and had to choose which of the same 2 buttons to press. This was randomly followed by the high or low reward (i.e., 50% contingency) according to **(B)** the temporal sequence. An “optimistic” response to this intermediate stimulus would therefore be to press the button that is paired with the high-reward (£4) option, whereas a “pessimistic” negative bias response would be the low-reward (£1) option. This response bias is designated p(mid as high), that is, the proportion of high-reward button presses for the intermediate stimulus. In the full version of the study (*n* = 990), participants demonstrated significant overall bias in **(C)** p(mid as high) and **(D)** drift rate.

The cognitive task (Figure 1A) had training and main task phases. For all trials, the stimulus was presented for 1000 ms, followed by a fixation cross for 750 ms (Figure 1B). Participants could respond from the beginning of stimulus presentation and were given feedback: “Correct, win £1” or “Correct, win £4” for 750 ms; or else “Timeout for incorrect response” for 3250 ms.

After pilot testing to minimize bias introduced by the visual stimuli (see the Supplement), two counterbalancing conditions were selected that showed minimal effect of counterbalancing condition on task performance. In one condition, high reward was paired with the vertical line and the ambiguous stimuli a line of 45° orientation; in the other, the high reward was paired with the horizontal line and the ambiguous stimulus a line of 135° orientation. In both versions the high reward was paired with the left-hand button. The participants were randomly but equally allocated one of the counterbalancing versions.

### Negative Bias Measures

The negative biases of the participants were measured in two overlapping ways. The first measure was the proportion of responses to ambiguous stimuli (in all cases this is a single stimulus that falls between the high and low exemplars and was the same for all participants) that were the same as the responses to the high-reward stimuli, that is, the proportion of high-reward responses to the intermediate cue, shortened to “p(mid as high)” hereafter. Given the contingencies for the intermediate stimuli, p(mid as high) = .5 would indicate no bias.

The second measure was the drift rate, calculated using the EZ drift diffusion model (20), which combines the choice proportion with the reaction times for high-reward choices on the ambiguous stimuli. This simplified version of the drift diffusion model (21) is strongly predictive of the full model (22) (see the Supplement).

### Statistical Analysis

All analyses were run in R (version 3.5.2) and all code, model specifications, and outputs are available online at https://github.com/ojr23/InterpretationBias.

#### Descriptive Analysis

We initially ran 1-sample *t* tests to determine if bias was significantly greater than 0.5 [p(mid as high)] or 0 (drift rate). Since the internal reliability of a measure puts an upper limit on its relationship with other measures, we also determined the split-half reliability (100,000 random splits using the splithalf function in the R psych toolbox) of responses to the intermediate trials. This measure should be >0.8 for the bias variables to be considered reliable. Following this, we performed a multivariate linear regression using the lavaan package to determine which questionnaire and demographic variables were significantly related to task performance. We used the lavaan “sem” function using maximum likelihood estimation with robust (Huber-White) standard errors for estimation, and all intercepts were modeled.

#### Model Comparison of Transdiagnostic Latent Variables

Next we explored the latent structure of the questionnaire measures. The linear regression assumes that the summary scores of the questionnaires represent discrete categories. However, it is possible that effects are driven by a transdiagnostic “mental ill health” factor. Or some questionnaires (e.g., BDI and trait anxiety, which are usually highly correlated) actually measure a single latent “negative affect” factor. We first explored 4 confirmatory factor analyses (CFAs) using the “cfa” function of the lavaan package and feeding the individual items from the questionnaires into 1, 2, or 3 latent factors (4 latent factors represents the 4 original questionnaires). We used maximum-likelihood estimation with robust (Huber-White) standard errors for estimation. Following this, we also entered all the individual items of the questionnaires into an exploratory factor analysis (EFA) and identified 3 latent factors using Cattell-Nelson-Gorsuch indices.

Having identified the 4-factor model as the most parsimonious (on the basis of lowest Akaike information criterion and Bayesian information criterion) as well as a good fit to the data (on the basis of root mean square error of approximation <0.08) we fed this latent factor structure into a new lavaan structural equation model (SEM) that combined this CFA with the original linear regression analysis on the task performance (including all the same demographic variables). This SEM again used maximum likelihood estimation with robust (Huber-White) standard errors.

We also entered all the individual items of the questionnaires into an EFA and identified 3 latent factors using Cattell-Nelson-Gorsuch indices (23). We then used these factor loadings in an exploratory SEM in which we set 3 latent variables (one representing each factor) and constrained the item-latent variable relationship to the weights extracted from the EFA.

#### Replication of Group Effect

To validate our findings, we attempted to recapitulate our original case-control group findings using the variables identified in the previous analysis steps. We did this in two ways. First, we used a theoretically motivated approach based on proposed cutoffs for the BDI questionnaire. Specifically, we used a cutoff of 29 (i.e., scores >28) to create a symptomatic depressed group (because it is a proposed cutoff for “severe” depression (24) and as such we thought it most likely to identify those who would meet criteria in a full clinical screening as in our prior study) and a score of <3 to create an asymptomatic control group. We did this using symptoms alone because that was how we screened individuals in our prior study. Second, we used a data-driven approach using multivariate latent mixture modeling. This was achieved using the MClust package in R. Specifically, maximum likelihood estimation was used to automatically test multiple specifications of latent Gaussian mixtures on BDI, IQ, and age (the significant measures from the SEM) and then identify the “winning” specification on the basis of Bayesian information criterion. Notably, we did not include task performance in our class enumeration so that classes are defined orthogonal to task performance. This approach identified 4 latent classes, and the groups with the highest and lowest bias scores were defined as the healthy and symptomatic groups, respectively. This approach attempts to cluster individuals into groups using features that are present in the data but may not be immediately obvious to the human eye (because they exist in complex multivariate space) or simple linear models. Thus, rather than doing a cutoff on BDI alone, all variables of interest are examined concurrently. Ultimately, we are using it as an exploratory way to divide individuals into groups so that we can recapitulate the group analyses from Aylward *et al.* (12) in a fully data-driven way.

## Results

All analyses are presented are available as a fully reproducible markdown script at https://github.com/ojr23/InterpretationBias. Demographic and questionnaire information are presented in Table 1. See the Supplement for further robustness checks (e.g., ruling out potential confounders).

### Bias

The split-half reliability of responses to the mid stimulus was high: λ = 0.92, 95% CI = 0.90–0.93. The full sample demonstrated p(mid as high) (test ≠ 0.5, *t*_989_ = 12, *p < .*001, 95% CI = 0.57–0.59) and drift rate (test ≠ 0, *t*_989_ = 12, *p < .*001, 95% CI = 0.003–0.005) that were significantly biased toward highest reward (Figure 1C, D).

### Linear Regression of Questionnaire Sum Scores

**Figure 2:**
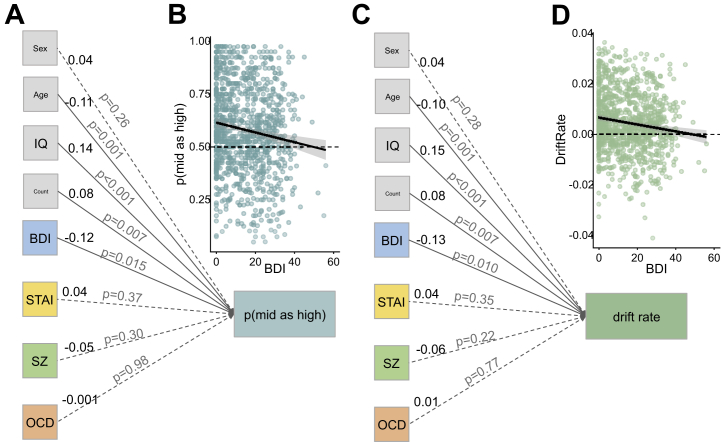
Linear regression on questionnaire summary scores demonstrates significant impact of depression (Beck Depression Inventory II [BDI]) symptomatology, IQ, age, and counterbalancing on **(A)** p(mid as high) task performance. We can illustrate the depression effect by **(B)** plotting raw BDI scores against raw task performance. The same pattern was seen when drift rate was **(C)** included in the regression and **(D)** plotted against raw task performance. The values in black to the right of each item are standardized regression coefficients (β); the value in gray on each line is the *p* value for the effect of that item on task performance; demographic variables are presented in gray boxes; psychiatrically relevant measures are in color; solid lines represent significant paths; dashed lines represent nonsignificant paths. OCD, obsessive-compulsive disorder, as measured with Obsessive-Compulsive Inventory-Revised; STAI, State-Trait Anxiety Inventory; SZ, Schizotypy short scale.

Affective bias and drift rate were both significantly influenced by IQ [p(mid as high): β = 0.14, *p <* .001; drift rate: β = 0.15, *p < .*001], age [p(mid as high): β = −0.11, *p = .*001; drift rate: β = −0.10, *p = .*001], BDI [p(mid as high): β = −0.12, *p = .*015; drift rate: β = −0.13, *p = .*001], and counterbalancing [p(mid as high): β = 0.08, *p = .*007; drift rate: β = 0.08, *p = .*007] only (Figure 2A, C). Thus, of mental health–relevant symptoms, task performance appears to be driven more by depression than by anxiety, obsessive-compulsive disorder (OCD), or psychosis-related traits*.* To illustrate the effect of depression in the regression we plotted the correlation between BDI and p(mid as high) (Figure 2B) and drift rate (Figure 2D) in raw data. Consistent with our prior work, increased depression is associated with reduced p(mid as high) (i.e., increased negative bias) on both of these plots. Note that when considered separately, STAI (*r* = −0.08, *p = .*018), Schizotypy short scale (*r* = −0.10, *p < .*001), and OCI-R (*r* = −0.09, *p = .*005) predict p(mid as high), but they do not do so over and above the effect of BDI (*r* = −0.13, *p < .*001).

### Latent Symptom Structure of the Questionnaires

**Figure 3:**
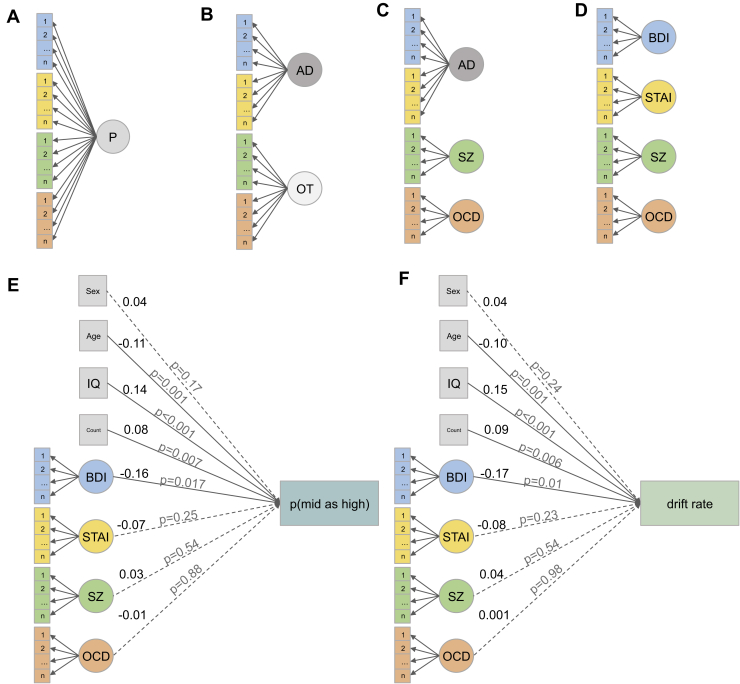
Full structural equation model (SEM) of symptoms and task performance. We first tested 4 confirmatory factor analyses (CFAs) of the latent structure of the symptom questionnaires: **(A)** a single psychopathology (P) factor model, **(B)** a 2-factor model comprising anxiety-depression (AD) vs. everything else (others [OT]); **(C)** a 3-factor model comprising AD vs. schizotypy symptoms (as measured with the Schizotypy short scale [SZ]) and obsessive-compulsive disorder (OCD) symptoms (as measured with the Obsessive-Compulsive Inventory-Revised); and **(D)** a full 4-factor model of each questionnaire. Following this we used the winning CFA **(D)** in a full SEM that combined the CFA with the initial regression. **(E)** Schematic representation (path diagram) representing the regression component only (see open data for full estimates) of the SEM for p(mid as high). **(F)** The same SEM for drift rate. The values in black to the right of each item represent standardized regression coefficients (β); the value in gray on each line is the *p* value for the effect of that item on task performance; demographic variables are presented in gray boxes; psychiatrically relevant measures are in color; solid lines represent significant paths; dashed lines represent nonsignificant paths; latent variables are represented as circles; observed variables are represented as boxes. BDI, Beck Depression Inventory II; STAI, State-Trait Anxiety Inventory.

We next tested whether the individual items from the questionnaires describe unique or transdiagnostic latent-symptom constructs. We tested 4 models (Figure 3A–D): a model in which there was a single generic psychopathology latent factor; a 2-factor model comprising an anxiety/depression factor versus everything else; a 3-factor model constituting an anxiety/depression factor, an OCD factor, and a schizotypy factor; and a 4-factor model comprising all 4 original questionnaires. The 4-factor model was the winning model (Table 2), which was substantially better [Bayes factor for the model (1) relative to the null (0)]; (BF_10_ = 656) than the next best 3-factor model as well as a good fit for the data (root mean square error of approximation < 0.08).

**Table 2:** Model Fits for the CFAs and SEM in Figure 3

CFA	BIC	AIC	RMSEA	RMSEA CI−	RMSEA CI+
P Factor (Figure 3A)	206,757	205,762	0.07	0.07	0.07
2-Factor (Figure 3B)	199,828	198,829	0.06	0.06	0.06
3-Factor (Figure 3C)	196,936	195,927	0.06	0.06	0.06
4-Factor (Figure 3D)	195,624	194,600	0.05	0.05	0.05
Full SEM					
p(mid as high) (Figure 3E)	199,538	198,357	0.052	0.052	0.053
Drift rate (Figure 3F)	193,869	192,689	0.052	0.052	0.053

AIC, Akaike information criterion; BIC, Bayesian information criterion; CFA, confirmatory factor analysis; CI−/+, negative/positive 95% confidence intervals; P factor, single psychopathology factor; RMSEA, root mean square error of approximation; SEM, structural equation model.

### Structural Equation Model Combining the Factor Structure With the Regression

We next combined the winning factor structure with the regression on task performance using an SEM (Figure 3E, F). Given that the 4-factor solution was the best fit, this is similar to the original sum score regression but allows the different items of the questionnaires to have varying influence over the latent factors. This full model suggests again that, of the psychiatrically relevant symptoms, it is depression symptoms alone that drive negative bias on this task [p(mid as high): β = −0.16, *p = .*017; drift rate: β = −0.17, *p = .*01]. Both models are also good fits to the data (Table 2).

### Exploratory Structural Equation Model

**Figure 4:**
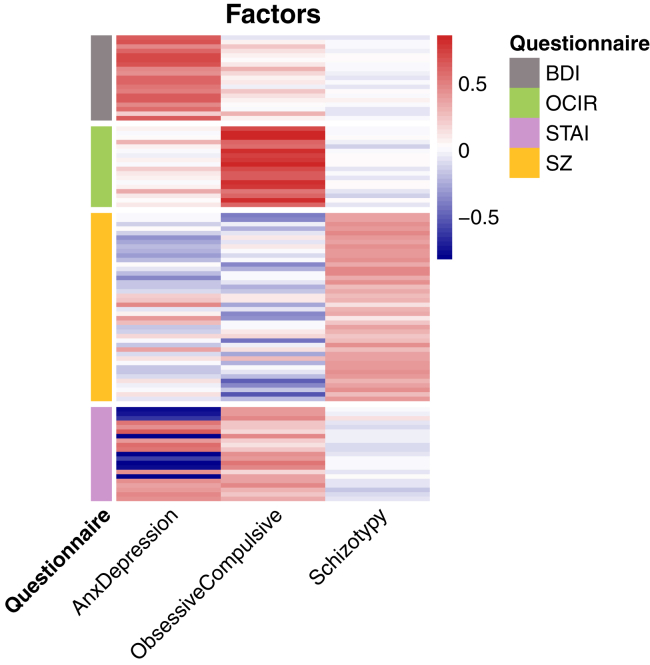
Heat map demonstrating factor loadings from the exploratory factor analysis of the original questionnaire items. BDI, Beck Depression Inventory II; OCIR, Obsessive-Compulsive Inventory-Revised; STAI, State-Trait Anxiety Inventory; SZ, Schizotypy short scale.

The EFA identified 3 factors (Figure 4), which we name “Anxiety-Depression,” because it maps closely onto the BDI and STAI, “Obsessive-Compulsive,” which is a mix of the OCI-R and STAI (and not the Schizotypy short scale), and “Schizotypy,” which loads positively almost exclusively on the Schizotypy short scale. In this exploratory SEM, we show that the Anxiety-Depression factor (F1) alone significantly influences task performance (see Table 3 comparing the EFA and CFA weights). This replicates the main inference from the paper, albeit collapsing depression and anxiety symptoms into a single latent factor. For full estimates and statistics see https://github.com/ojr23/InterpretationBias.

**Table 3:** *p* Values and Standardized Regression Weights From the Regression Component of the Exploratory SEM Compared With Confirmatory SEM Predicting p(mid as high)

Exploratory SEM			Confirmatory SEM		
Variable	*p*	β	Variable	*p*	β

Counterbalance	.007	0.086	Counterbalance	.008	0.083
IQ	<.001	0.145	IQ	<.001	0.143
Age	<.001	−0.115	Age	.001	−0.109
Sex	.244	0.038	Sex	.187	0.043
Anxiety-Depression	.012	−0.094	BDI	.017	−0.161
			STAI	.268	−0.072
Obsessive-Compulsive	.26	−0.045	OCI-R	.877	−0.008
Schizotypy	.82	0.008	SZ	.547	0.037

BDI, Beck Depression Inventory II; OCI-R, Obsessive-Compulsive Inventory-Revised; SEM, structural equation model; STAI, State-Trait Anxiety Inventory; SZ, Schizotypy short scale.

### Recapitulation of Original Group Effect

#### Symptom Cutoffs

**Figure 5:**
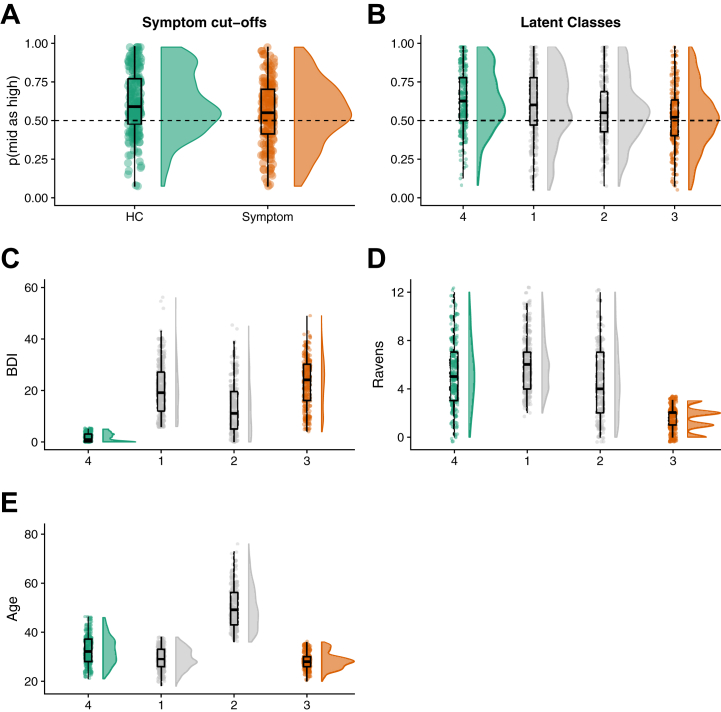
We recapitulated prior group effects using **(A)** symptom cutoffs on depression symptoms and **(B)** latent class analysis. In both cases, groupings are created using symptom characteristics [as well as demographic characteristics in **(B)**] but demonstrate clear group effects in task bias scores (which are not used for group assignment). Latent class analysis also shows that in the optimal 5-group solution, groups differ according to **(C)** depression symptoms, **(D)** IQ, and **(E)** age. BDI, Beck Depression Inventory II; HC, healthy control.

We defined control individuals as those with BDI < 3 (*n* = 197) and symptomatic individuals as those with BDI > 28 (*n* = 170). There was a significant main effect of group on p(mid as high) (*t*_348_ = 2.8, *p = .*005, *d* = 0.3; Figure 5A).

#### Latent Mixture Modeling

Latent mixture modeling on the IQ, BDI, and age variables (only) identified a winning model (Bayesian information criterion = −19,367) comprising 4 latent classes (*n* = 297, 219, 246, and 228) with a diagonal distribution and varying class volume and shape. Notably, randomizing the initialization of the modeling and fitting it 1000 times identified this 4-class solution 90% of the time (with the next most common 5-factor solution 6% of the time). We then ordered these classes by mean p(mid as high) score (which was not used for class enumeration and so is orthogonal to class definition) and defined the highest and lowest bias groups as asymptomatic and symptomatic, respectively. There was a significant group difference in bias between these groups [p(mid as high) by group: *t*_534_ = 4.5, *p < .*001, *d* = 0.41] (Figure 5B), meaning that the task performance extremes of these groups (determined by symptoms) do significantly differ on task performance. Confirming our initial study, the group with lowest p(mid as high) score (i.e., greatest negative bias) also had the highest mean depression scores, while those with the highest p(mid as high) score had low depression scores (Figure 5C). Interestingly, the “symptomatic” latent class is also particularly of low IQ (Figure 5D) relative to the other classes, but both symptomatic and asymptomatic are approximately the same age (Figure 5E).

## Discussion

Individual differences in interpretation bias on this fully translational task were shown to be reliable and linked to individual differences in depression symptoms in a large unscreened sample. Thus, we have uniquely bridged both the group-to-individual and animal-human generalizability gaps for a measure of negative affective bias. Moreover, we have shown that task performance is also related to age, IQ, and stimulus counterbalancing.

The primary inference from the CFA is that performance on this translational interpretation bias task is most clearly related to individual differences in depression symptomatology than to any other psychiatrically relevant symptom (although note that the exploratory approach suggests that anxiety and depression scales may be largely measuring the same construct). Thus, the effects in our previous case-control study comparing individuals with mood and anxiety disorders versus control individuals (12) may have been driven more by depression than by anxiety symptoms. Indeed, using depression symptoms to create groupings (either with symptom cutoffs or latent mixture models) can recapitulate our prior group findings.

An important question, therefore, is why this task might be more sensitive to depression than anxiety, which also is associated with negative affective biases (25). One explanation is that our experiment was conducted using rewards and that depression is more associated with reward deficits (26) than anxiety [which may be more related to threat processing (25)]. Future work could seek to further identify the specific symptoms of depression that are affected (e.g., loss of motivation, reward insensitivity) using more specific symptom scales. Of course, it is also important to note that when considered separately, the other symptom scales also correlate with bias, just not over and above the effect of depression when considered together. Researchers should therefore exercise caution when interpreting the correlation of single-symptom scales with cognitive task measures.

Critically, we also demonstrate that a measure of negative bias used in rodents can be extended to humans with clinical diagnoses (12) and then also adapted for online studies to gather large samples of participant data. It is essential to create more measures that span human and rodent research, given the lack of clear human analogues for animal models of depression and associated difficulties in treatment-development pipelines (12). Having a directly translatable task will enable us to more rapidly screen the effects of candidate interventions on symptoms of interest as well as potentially further our understanding of the neural mechanisms of negative biases in more invasive rodent work. Critically, future work can now determine if similar neural mechanisms underlie task performance across species.

It is worth noting that this task is also sensitive to age and IQ. Specifically, older individuals and those with lower IQ are more likely to demonstrate negative bias on this task over and above any depression symptomatology. Interestingly, this age effect is inconsistent with a commonly observed increase in “positivity” bias as individuals get older (27), but not with the observation that older age is a risk factor for depression (28). Our findings are also consistent with the increased risk for depression in individuals with lower IQ (29). Ultimately this means that for this task to be used clinically, it would be important to control for these factors when personalizing treatment decisions on the basis of task performance. Indeed, it is highly likely that many of the cognitive tasks that we develop in the mental health field are also sensitive to such characteristics (23), but it is only through large-scale testing (and development of sensitive between-subject measures) that we can begin to identify them.

There are limitations to this study that should be highlighted. First, ∼7% of the participants did not adequately complete the cognitive task. Previous online experiments have minimized this problem through testing participants between training and the main task, and providing more training if they fail (23). The task could also have benefited from probe questions to test attention and tests for implausibly fast reaction times (23), as well as ensuring that participants understood instructions (30). It should also be noted that there were no exclusion criteria in this study, aside from failure to complete the cognitive task. This was by design, because we wanted to cast as wide a net as possible. Mechanical Turk samples have been shown to be very similar to unscreened representative samples (31), and it has been shown that participants in online experiments demonstrate behaviors consistent with those found in laboratory studies (32). Nevertheless, to demonstrate true generalizability, it will be important to replicate this effect in different samples. Notably, the mean self-report scores on the BDI and the OCI-R were high for an online sample (and the OCI-R data appeared bimodal), potentially indicating an unusually pathological sample (e.g., 47% of our sample met a proposed clinical cutoff of 21 on the OCI-R, and 16% met a cutoff of 29 on the BDI). One major reason for this may be that our dataset was not restricted to a U.S. sample and there are cultural differences in the interpretation of certain symptoms (especially OCD symptoms—see the Supplement for comprehensive analysis and discussion). Nevertheless, future work should introduce more comprehensive validity checks on the questionnaires to minimize potential noise. Similarly, the latent profile analysis also should be considered exploratory until replicated in another sample. It demonstrates that it may be possible to recapitulate prior group studies in unscreened samples, but the specific profiles warrant replicating. On a related note, now that we have shown the task to be sensitive to symptoms, it may be possible to set a task performance threshold that can be used to determine who might be a candidate for specific treatments. This can then be used to determine the sensitivity and specificity of the task for group assignment.

It should also be noted this study was not designed to make claims about the directionality of the relationship between negative biases and depression, which would require longitudinal analyses. However, establishing that this task is sensitive to individual differences as well as possessing good split-half reliability is a prerequisite for such future longitudinal studies, so this paper can form the foundation for such research in clinical trials (although future work should also explore reliability over longer time scales, such as test-retest reliability). Moreover, uniquely demonstrating relevance of the scale across humans and animals establishes its value for treatment development regardless of the underlying directionality. It is also possible that the different variables (e.g., age and depression) moderate each other. Given that we have no a priori predictions about this we do not test and build such models, however our data are available online for future exploratory analyses.

Another important limitation is that despite efforts to minimize the bias introduced by different counterbalancing conditions, the regression analyses indicate that the two remaining counterbalancing conditions still had a significant effect on task performance. Future studies may consider using a single stimulus set. More broadly, however, this illustrates a serious issue with translating group-level findings into individualized predictions. Most cognitive neuroscience in the mental health domain uses group studies to determine efficacy of interventions or identify potential biomarkers. As we move toward a more personalized-medicine approach, it cannot be taken for granted that it will be easy to make the transition from group-level findings to measures of individual differences (33). It is therefore critically important to demonstrate that cognitive measures are able to bridge this group-individual generalizability gap.

A final caveat is that the inferences from the confirmatory and EFAs are slightly different. In the confirmatory approach, we did not find support for a model that combined all the items across depression and anxiety scores, whereas in the exploratory approach they (largely) combined into a single factor. One reason for this is that some of the individual items in these questionnaires also load onto other factors (e.g., some STAI items load more heavily onto a factor with OCD symptoms), which the exploratory approach is able to identify. Future work can use these exploratory results to refine predictions for confirmatory approaches.

### Conclusions

Individual differences in interpretation biases may provide a nonsubjective measure of the severity of depressive symptoms. To the best of our knowledge, this is the first demonstration of a cognitive measure of negative affective bias that spans both the group-individual and animal-human generalizability gaps. Thus, we can track a single task and psychiatrically relevant symptoms from preclinical rodent work (11), to groups with clinical diagnoses (12), and to a measure of individual differences at scale.
