# Supplementary material for: Association Between a Directly Translated Cognitive Measure of Negative Bias and Self-reported Psychiatric Symptoms
Source: Biol Psychiatry Cogn Neurosci Neuroimaging. 2022 Feb;7(2):201–9. doi: 10.1016/j.bpsc.2020.02.010 (PMC8816734; doi:10.1016/j.bpsc.2020.02.010)
Supplement: Supplemental Material [file mmc1.pdf]

Association Between a Directly Translated Cognitive Measure of Negative Bias and Self-reported Psychiatric Symptoms

Supplementary Information

Previous Findings

Figure S1: This study builds on prior work developing a measure of negative affective bias as indexed by proportion of mid tones interpreted as high reward (‘p(mid)as high’) in A) a rat pharmacological model of mood and anxiety disorders and B) humans with mood and anxiety disorders relative to healthy controls. Specifically, symptomatic ('Symptom') rats and humans both demonstrate significantly increased negative affective bias (i.e. reduced prediction that ambiguous outcomes will lead to higher rewards) relative to non-symptomatic controls (‘HC'). The effect size of the Human group difference is d= 0.72.

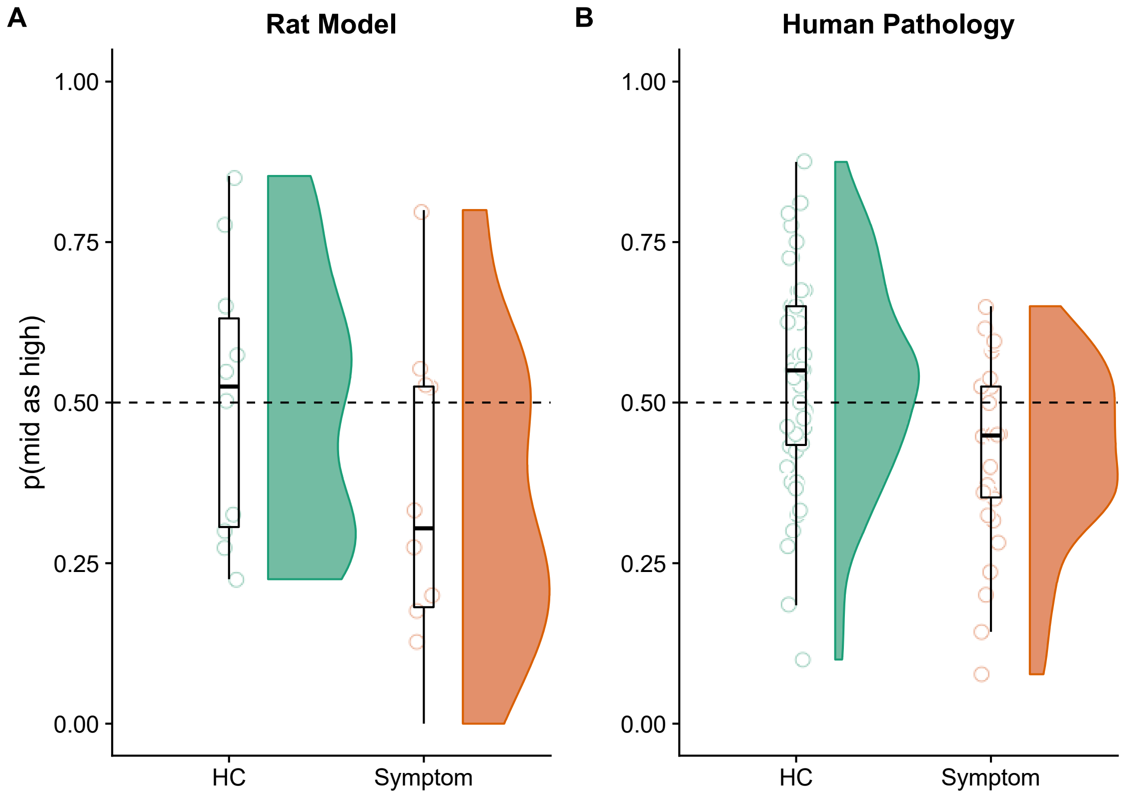

Pilot

Prior to the main study, extensive piloting was carried out to minimise the amount of bias introduced by the stimuli used to translate the task into the visual domain (**figure S2**). Pilot 1 (circle size) had 4 counterbalancing versions based on the stimuli, response and outcomes (see **table S1** for full details). Following discovery of clear between-subject bias, pilot 2 used line orientation and had 8 counterbalancing versions (see **table S2**). Further between-subject bias meant the main study was restricted to two counterbalancing versions from pilot 2.

Results

Both pilots demonstrate clear sources of between-subject bias (**figure S2**; main effect of counterbalancing in pilot 1 ($F_{(3,260)}$=35,p<0.001) and pilot 2 ($F_{(7,143)}$=3,p=0.005). Individuals demonstrated 'higher' bias when large (or vertical) stimuli were paired with large rewards on the right-hand side. These likely reflect pre-potent biases (e.g. bigger sizes are associated with numerically higher amounts and in Latinate languages we read from left to right). For the main task, testing was therefore restricted to counterbalancing 1 and 7 from pilot 2 as this constituted the smallest difference between two counterbalancing conditions across the two pilots (mean difference=0.003, $p_{(Tukey)}$=1). Pilot 2 design was also preferred over pilot 1 because there is only one interpretation of line orientation, whereas a circle has both area and diameter.

Table S1: Visual representation of the different counterbalancing conditions in Pilot 1, demonstrating the high and low rewarding stimuli, and the key responses required.

**Condition**	**1**	**2**	**3**	**4**
**High reward**	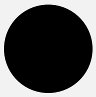	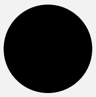	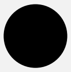	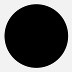
**Response**	Z	M	Z	M
**Low reward**	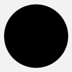	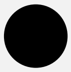	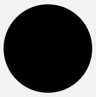	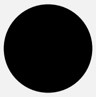
**Response**	M	Z	M	Z

Table S2: Visual representation of the eight different counterbalancing conditions in pilot 2, demonstrating the stimuli associated with high and low reward, the key response required, and the ambiguous stimulus used.

**Condition**	**1**	**2**	**3**	**4**	**5**	**6**	**7**	**8**
**High reward**								
**Response**	Z	M	Z	M	Z	M	Z	M
**Low reward**								
**Response**	M	Z	M	Z	M	Z	M	Z
**Ambiguous stimulus**	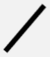	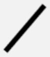	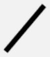	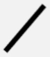	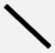	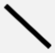	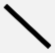	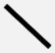

Figure S2: **Task schematic and development.** Participants A) completed two versions of the affective bias task during piloting (P1= pilot 1, which used circles and P2= pilot 2 which used lines; see supplement for key to the numbered counterbalancing conditions). They were first trained on 'extreme' cues (large or small circles in P1 or vertical or horizontal lines in P2) that had 100% contingencies with high or low reward (hypothetical £1 or £4) contingent on participants pressing the correct button (left or right; represented as blue or yellow in this figure). Following training, participants were presented with intermediate stimuli (mid size circle in P1 or angled lines in P2) and had to choose which of the same two buttons to press. This was randomly followed by the high or low reward (i.e. 50% contingency). An 'optimistic' response to this intermediate stimulus would therefore be to press the button which is paired with the high reward option (£4), whereas a 'pessimistic' negative bias response would be the low reward (£1) option. This response bias is designated p(mid as high) – i.e. the proportion of high reward button presses for the intermediate stimulus. The stimulus presentation durations were the same across all trial types (represented pictorially at the bottom of 2A). The stimulus-response-outcome mappings led to different counterbalancing versions which B) showed clear counterbalancing effects on p(mid as high) in the circle version (P1; N=264) and in the C) line version (P2; N=158). The specific mappings referred to by the x-axis numbers are presented in the supplement but here ordered by magnitude of bias.

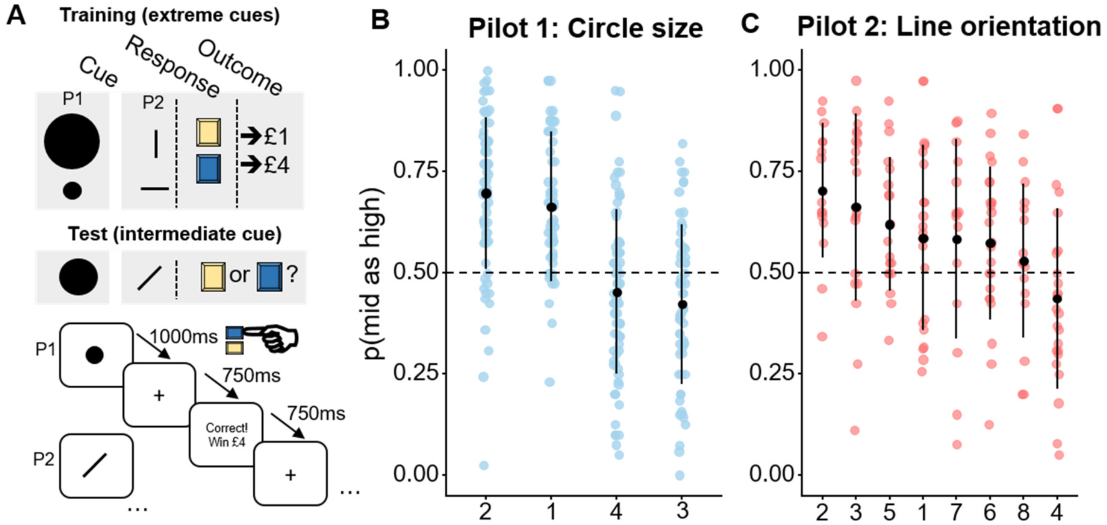

Supplemental Questionnaire Details

We included only the Trait component of the STAI in all subsequent analyses as it is designed to capture stable trait symptoms (but is also highly correlated with the State measure). The Beck Depression Inventory was presented without the 9th item concerning suicidal thoughts as we did not wish to solicit information about suicidal thoughts without the ability to intervene. The OCI-R and SZ measures were chosen as negative controls for the mood and anxiety disorder questionnaires (i.e. to demonstrate effects were specific rather than generic), as well as to be consistent with previous research examining identifying individual differences in pathology (1). A 12-item form of Raven's Progressive Matrices (2) was completed as a measure of IQ as it is strongly predictive of (0.90) the full 36-item Advanced Progressive Matrices (2), but is short enough to minimize participant drop-out. Participants were also required to fill out demographic questionnaires indicating their age, gender, as well as various mental-health related questions.

Supplemental Task Details

The training phase used 'extreme' cues only, and participants were instructed to maximize the amount earned in a 2-alternative-forced-choice task. Participants pressed a button (left or right) when they saw the upper and lower extreme stimuli (e.g., high and low frequency tones in the original task; vertical/horizontal lines in this task) to receive a reward (£1 or £4). The stimulus-response-outcome contingencies were 100% (but counterbalanced across individuals) and acquired by participants on a trial and error basis. In the main task, participants were also presented with intermediate stimuli (diagonal line) which was randomly reinforced with the two reward outcomes (i.e., a contingency of 0.5). The test version of the task had 40 trials for each intermediate/extreme stimulus (total=120).

Supplemental Modelling Details

The DDM models decision making as a process of evidence accumulation towards a decision threshold, utilising speed and accuracy of responses to model the biases that shape our responses. The parameter of interest here (drift rate) indicates the rate of information accumulation towards a response (3), and has been shown to be more negative in individuals with mood and anxiety disorders (4). No bias on this measure would be driftrate=0. As previous studies showed no evidence of prior bias (i.e.

'starting point') when using this task (4), we used the EZ diffusion model which assumes no bias (and which is computationally tractable with this number of participants).

Supplemental Results

Missing data

The reported parameters are obtained using list-wise deletion of the individuals with incomplete data (N=70: final sample N=990). See **table 1** for comparison across included/excluded individuals. However, case-wise maximum likelihood estimation of missing data results in identical inference (albeit slightly stronger effect-size for the effect of depressive symptoms) in the full sample (N=1060; for estimates see: https://github.com/ojr23/InterpretationBias).

Confounds

To confirm robustness to confounds, we also re-ran the initial regression analysis but added answers to questions about past, current or family history of diagnoses or symptoms as well as current treatment use/seeking. This model resulted in identical inference to the model of interest and none of these additional factors were significantly related to task performance indicating that these factors do not confound the observed symptom effects (see supplementary **table S3** and https://github.com/ojr23/InterpretationBias).

To exclude potential bots we also reran analyses excluding individuals with mean task RTs 2 standard deviations below the mean for the low stimulus (N=36 excluded). When we do this the statistical inference remains identical (see https://github.com/ojr23/InterpretationBias). Moreover, there are no individuals who have an accuracy difference of 1 for the high vs low trials (i.e. individuals who are bashing a single key for every trial).

Table S3: p-values and standardized regression weights from a regression including potential symptom, treatment or diagnosis confounds

	p	ß
Sex	0.121	0.05
Age	0.001	-0.107
IQ	0.001	0.133
Counterbalance	0.008	0.082
BDI	0.016	-0.124
Trait STAI	0.667	0.022
SZ	0.366	-0.049
OCIR	0.702	0.019
Past Mental Health	0.816	-0.01
Current Mental Health	0.42	0.033
Sought Help for Mental Health	0.095	0.071
Currently Undergoing Treatment	0.434	-0.038
Currently Taking Medication	0.903	0.006
Family Hist. Schizophrenia.	0.197	-0.053
Family Hist. Bipolar	0.478	-0.026
Schizophrenia	0.111	0.073
Bipolar	0.428	-0.033
Neurological Disorder	0.596	-0.02
Take Illicit Drugs	0.444	0.027
Drink Alcohol	0.528	-0.02
Learning Disability	0.748	-0.012

Bimodality in OCIR data

One perplexing issue is the presence of bimodality in the OCIR scale (**Figure S3a**) which leads to substantially higher mean symptoms than other studies (for example (1)). Indeed, in our sample 499 participants out of the 1060 met a clinical cut-off of 21 for OCD (5), which seems unusually high. In a post-hoc exploratory analysis, however, we identified substantially higher distributions for the OCIR sum scores for those tested in the UTC+05:00 (Pakistan and Central Asian -stans) and UTC+05:30 (India and Sri Lanka only; **Figure S3b**) timezones. As such, it appears that these timezones are driving the bimodality.

Figure S3: **OCIR data** demonstrates A) substantial bimodality. This is B) likely driven by individuals in the +5 (Pakistan/ Central Asia) and +5.5 (Indian/Sri Lankan) time zones (who have been excluded from many previous online studies using this scale).

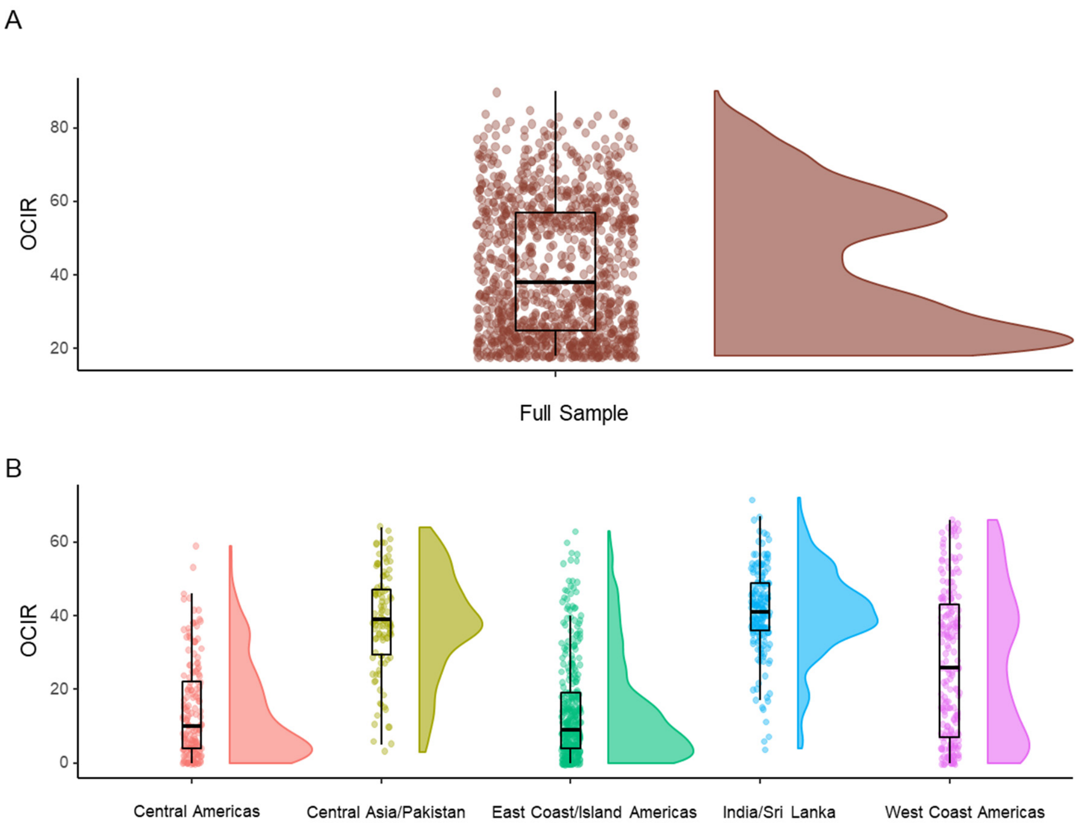

In prior studies (e.g. (1)) recruitment has often been restricted to the USA. For direct comparison, in our samples from within USA time zones, the mean OCIR scores fall well below 21 (**Table S4**), with the exception of the west coast of the Americas which displays some bimodality (**Figure S3b**; interestingly

Califorina saw the the highest proportion of Indian migrants in the US between 2014-2018 (6)). Notably, prior work has demonstrated considerably higher scores on the OCI-R in individuals of Asian relative to White or Black heritage, which has been speculated to reflect cultural differences in how OCD symptoms are interpreted and reported (7). Thus, we argue that this bimodality may be due to the uniquely global nature of our sample and reflect the impact of cultural and/environmental factors on questionnaire responses.

Table S4: **Mean OCIR scores as a function of UTC (Universal Coordinated Time) Time zone**. The geographical area is an indicative summary of the most populous areas and not exhaustive list. Note we have removed rows with fewer than 5 individuals to avoid compromising anonymity. N=number of cases, S.D.= standard deviation.

UTC Time zone	Geographical area	N	Mean	S.D.
-7	West coast Americas	199	26.7	20.1
-6	Americas Mountain time	17	16.5	16.4
-5	Central Americas	164	14.2	13.3
-4	East Coast Americas/Islands	336	14.2	14.1
-3	South America	6	18.2	13.0
1	UK/Ireland	14	16.1	11.9
2	Mainland Europe/Central Africa	10	18.2	13.6
3	Russia/Turkey/East Africa	23	23.9	16.2
5	Central Asia/Pakistan	103	38.2	14.0
5.5	India/Sri Lanka	174	41.1	11.6
8	China/Southeast Asia	6	12.8	8.9

It is also worth noting that time zone also had an effect on both depression and anxiety scores, but it had a much larger effect on OCIR scores than any other variable (**Figure S4**). Critically, including time zone in the primary regression analysis had no impact on the inference (and time zone was not itself a significant predictor of task performance **Table S5**). Thus, while time zone accounts for considerable variance in symptom scores, it does not influence the relationship between symptoms and cognitive task performance. Overall, this exploratory analysis highlights the importance of considering cultural/environmental predictors in psychiatry research and the importance of trying to generalize outside of WEIRD (Western, Educated, Industrialized, Rich, and Democratic) samples (8).

Figure S4: Regression weights from a linear regression model predicting time zone (Timezone ~ GenderMF + Age + Ravens + spreadsheet +BDI + STAI2 + SZ + OCIR + propmedhigh) demonstrating the biggest impact of OCIR scores (and no impact of task performance (propmedhigh)).

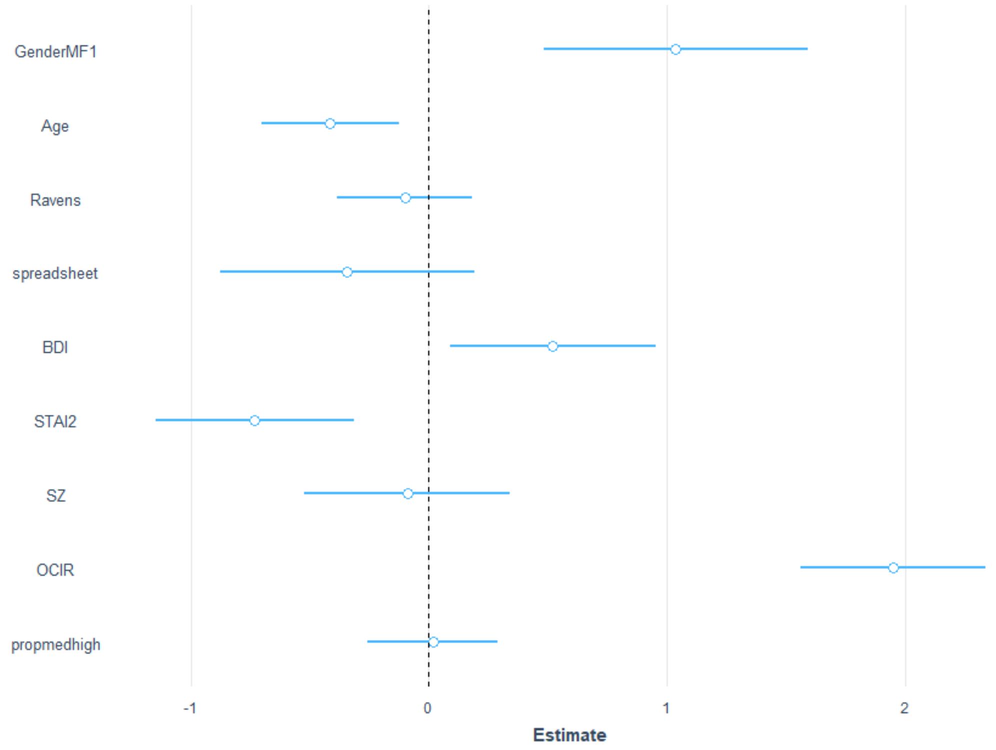

Table S5: Including time zone in the primary regression analysis leads to identical inference and time zone is not a significant predictor of task performance.

	p	β
**Counterbalance**	0.006	0.085
**IQ**	<0.001	0.144
**Age**	<0.001	-0.112
**Sex**	0.2	0.041
**BDI**	0.015	-0.124
**STAI**	0.338	0.045
**OCD**	0.988	-0.001
**SZ**	0.246	-0.057
**Time Zone**	0.905	0.004

Supplemental Discussion

It is also worth noting that the task developed here bears some resemblance the probabilistic reward task developed by Pizzagalli and colleagues (9). In this prior task, two stimuli (a short or long line) are reinforced (with the same 5c reward) either sparingly or frequently. In other words, subjects learn over time that one stimulus is better because it is more frequently rewarded. Depressed individuals fail to show a bias towards the more frequently rewarded stimulus. This is similar to our effect in that the more depressed individuals show less of a bias towards the more highly rewarded stimulus. However, the Pizzagalli task requires individuals to integrate rewards over time (i.e. learn) whereas in our current task the feedback is asymmetric on each trail (£1 vs £4) and the main version of the task occurs after learning of the high and low exemplars has already taken place. Of course, the 50% contingencies of the ambiguous stimuli are still reinforced at 50% in our task, which means that they should eventually learn to respond at 50%. Our overall bias suggests that they don't and, moreover, the high split half reliability we see means that performance is consistent over time and that learning differences on these ambiguous stimuli are unlikely to be driving the effect on our task. Of note, work using reinforcement learning models and the Pizzagalli task (10) also indicate that effects may be more driven by differential *sensitivity* to reward in depression than *learning rates* (i.e. depressed individuals are less motivated by rewards, rather than being slower to learn about them). As such performance across both tasks may be driven by the same underlying mechanisms. Future work should seek to collect data from both tasks in the same individuals to determine how related performance across these tasks is.

Also, while the task design being directly translated from animal models is a key strength of our study, the limitations of this method should also be noted. Our stimuli were notably abstract, whereas negative biases are often seen in self-relevant stimuli (11). As a result, this task could be more of a test of cognitive performance rather than affective bias (hence the correlation with IQ). Future work could control for this by including a cognitive task with no affective component (e.g. the same task with no reinforcement) as a control condition.

Supplemental References

1. Gillan CM, Kosinski M, Whelan R, Phelps EA, Daw ND. (2016). Characterizing a psychiatric symptom dimension related to deficits in goal-directed control. *ELife*. 5:e11305

2. Arthur W Jr, Day DV. (1994). Development of a short form for the Raven Advanced Progressive Matrices test. *Educ Psychol Meas*. 54:394-403.

3. Ratcliff R, McKoon G. (2008). The diffusion decision model: Theory and data for two-choice decision tasks. *Neural Comput*. 20:873–922.

4. Aylward J, Hales C, Robinson E, Robinson OJ. (2019). Back-Translating a rodent measure of negative bias into humans: the impact of induced anxiety and unmedicated mood and anxiety disorders. *Psychol Med*. Jan:1-10.

5. Foa EB, Huppert JD, Leiberg S et al. (2002). The obsessive-compulsive inventory: development and validation of a short version. *Psychol Assess*. 14:485-496.

6. Retrieved from: migrationpolicy.org/programs/data-hub/charts/us-immigrant-population-state-and-county on 20/02/2020

7. Wu KD, Wyman SV. (2016). Examination of racial differences in assessment of OCD symptoms and obsessive beliefs. *J Obsess Compul Relat Disord*. 10:10-18.

8. Henrich J, Heine SJ, Norenzayan A. (2010). The weirdest people in the world? *Behav Brain Sci.* 33:61-83.

9. Pizzagalli DA, Iosifescu D, Hallett LA, Ratner KG, Fava M. (2008). Reduced hedonic capacity in major depressive disorder: Evidence from a probabilistic reward task. *J Psychiatry Res.* 43:76–87

10. Huys QJ, Pizzagalli DA, Bogdan R, Dayan P. (2013). Mapping anhedonia onto reinforcement learning: a behavioural meta-analysis. *Biol Mood Anxiety Disord.* 3:12.

11. Beck A. (1987). Cognitive models of depression. *J Cogn Psychother*. 1: 7–37.
